# Two homozygous adjacent novel missense mutations in DYSF gene caused dysferlinopathy due to splicing abnormalities

**DOI:** 10.3389/fgene.2024.1404611

**Published:** 2024-06-05

**Authors:** Lun Wang, Yan Zhou, Tiantian Wei, Hongyao Huang

**Affiliations:** ^1^ Jinzhou Medical University Graduate Training Base, Suizhou Central Hospital Affiliated to Hubei University of Medicine, Suizhou, Hubei, China; ^2^ Department of Basic Medicine, School of Medicine, Jingchu University of Technology, Jingmen, Hubei, China; ^3^ Daytime Surgical Ward, Suizhou Hospital, Hubei University of Medicine, Suizhou, Hubei, China; ^4^ Department of Laboratory, Suizhou Hospital, Hubei University of Medicine, Suizhou, Hubei, China

**Keywords:** Dysferlinopathy, DYSF, splicing, WES, minigene, homozygous

## Abstract

**Background:** Dysferlinopathy is an autosomal recessive disorder caused by mutations in the DYSF gene. This study reported two homozygous adjacent missense mutations in the DYSF gene, presenting clinically with bilateral lower limb weakness and calf swelling. Two homozygous adjacent missense mutations in the DYSF gene may be associated with the development of dysferlinopathy, but the exact mechanism needs further investigation.

**Methods:** A retrospective analysis of clinical data from a dysferlinopathy-affected family was conducted. Peripheral blood samples were collected from members of this family for whole-exome sequencing (WES) and copy number variation analysis. Sanger sequencing was employed to confirm potential pathogenic variants. The Human Splicing Finder, SpliceAI, and varSEAK database were used to predict the effect of mutations on splicing function. The pathogenic mechanism of aberrant splicing in dysferlinopathy due to two homozygous adjacent missense mutations in the DYSF gene was determined by an *in vivo* splicing assay and an *in vitro* minigene assay.

**Results:** The proband was a 42-year-old woman who presented with weakness of the lower limbs for 2 years and edema of the lower leg. Two homozygous DYSF variants, c.5628C>A p. D1876E and c.5633A>T p. Y1878F, were identified in the proband. Bioinformatics databases suggested that the mutation c.5628C>A of DYSF had no significant impact on splicing signals. Human Splicing Finder Version 2.4.1 suggested that the c.5633A>T of DYSF mutation caused alteration of auxiliary sequences and significant alteration of the ESE/ESS motif ratio. VarSEAK and SpliceAI suggested that the c.5633A>T of DYSF mutation had no splicing effect. Both an *in vivo* splicing assay and an *in vitro* minigene assay showed two adjacent mutations: c.5628C>A p. D1876E and c.5633A>T p. Y1878F in the DYSF gene leading to an Exon50 jump that resulted in a 32-aa amino acid deletion within the protein. Point mutation c.5628C>A p. D1876E in the DYSF gene affected splicing *in vitro*, while point mutation c.5633A>T p. Y1878F in the DYSF gene did not affect splicing function.

**Conclusion:** This study confirmed for the first time that two homozygous mutations of DYSF were associated with the occurrence of dysferlinopathy. The c.5628C>A p. D1876E mutation in DYSF affected the splicing function and may be one of the contributing factors to the pathogenicity.

## Introduction

Dysferlinopathy encompasses a category of neuromuscular diseases characterized by the absence of dysferlin in the skeletal muscle. It is an autosomal recessive disorder (Urtizberea et al., 2008) caused by mutations in the DYSF gene encoding the dysferlin protein (Park et al., 2023). Dysferlin is a 230-kDa protein belonging to the ferlin family of proteins containing seven calcium-binding C2 structural domains associated with calcium-dependent membrane fusion and repair (Glover and Brown, 2007). Detection of ferlin deficiency in muscle or blood and identification of mutations in the DYSF gene are the main tools for diagnosing dysferlinopathy (Fanin and Angelini, 2016). DYSF mutations are associated with various phenotypes, including asymptomatic creatine kinase elevation and selective, progressive involvement of proximal and/or distal muscles of the limbs. The two most predominant dysferlinopathy phenotypes are limb-girdle muscular dystrophy type 2B (LGMD2B) and Miyoshi muscular dystrophy-1 (MMD1). The former manifests mainly as proximal weakness of the lower limbs, whereas the latter manifests as a distal myopathy that progressively affects the posterior hamstring compartment muscles of the leg (Arab et al., 2023; Park et al., 2023). Because these phenotypes are close to and easily confused with other causes of muscular dystrophy, clinical diagnosis of dysferlinopathy requires a combination of isoferrin deficiency in the blood and genetic testing to detect DYSF mutations. To date, only a few cases of DYSF mutations have been reported worldwide (Illa et al., 2001; Sinnreich et al., 2006; Therrien et al., 2006), and functional validation of the pathogenicity of mutations is rare.

In recent years, with the in-depth study of dysferlinopathy, scholars worldwide have also conducted a series of studies on mutation pathogenicity. Liu constructed a candidate region spanning Miyoshi myopathy. This study identified five skeletal muscle ESTs mapping to this region. One EST was located in a new full-length 6.9-kb muscle cDNA, and the corresponding protein was named dysferlin (Liu et al., 1998). By studying autosomal recessive limb-girdle muscular dystrophy type 2 (LGMDR2), Bashir cloned a partial cDNA of dysferlin. The protein comprises 1779 amino acids and contains two C2 structural domains and a C-terminal transmembrane structural domain. Northern blot analysis detected approximately 7 kb of transcripts in the skeletal muscle, heart, and placenta. Weaker transcripts were detected in the liver, lung, kidney, and pancreas. A 7-kb transcript was detected in the cerebellum and medulla oblongata. A 4-kb transcript was detected in all brain regions except the spinal cord, with the highest expression level in the nucleus accumbens. No dysferlin was detected in the fetal brain tissue (Bashir et al., 1998).

Seo K constructed a knock-in dysferlinopathy mouse model for the p. Q832* nonsense mutation in DYSF (Seo et al., 2021). The constructed purist mice lacked the dysferlin protein in the skeletal muscle. After 2 weeks of oral ataluren treatment, abnormal dysferlin protein expression was restored, skeletal muscle pathology improved, and various physical functions of the mice also improved. This study confirmed the applicability of ataluren treatment to dysferlinopathy patients with nonsense mutations in DYSF. Schoewel et al. (2012) suggested that point mutations in DYSF leading to misfolding, endoplasmic reticulum aggregation, and amyloid formation are responsible for LGMD2B. Short peptides derived from dysferlin and labeled as the cell-penetrating peptide TAT were designed to validate this theory. It was then confirmed by laser damage assay and interventional atomic force microscopy that the mutant dysferlin protein restored membrane repair function in primary human myotubes from patient adult myoblasts.

Notably, in addition to functional consequences at the protein level, missense variants can also have deleterious effects by affecting the normal splicing of pre-mRNA (PMID: 11967553). Altered pre-mRNA splicing is currently thought to be the underlying cause of many hereditary diseases (PMID: 26593421, PMID: 21750108). Possible splicing defects caused by missense mutations in the DYSF gene were assessed by transcriptional analysis of patient blood samples and the minigene splicing reporter system. This study reported a family with suspected dysferlinopathy in which the preexisting patient had bilateral lower limb weakness and a calf swelling phenotype. Two adjacent *de novo* missense variants were identified in the DYSF gene of this patient by whole-exome sequencing (WES), but the mutations were of undetermined significance. In order to investigate the pathogenicity of this mutation, this study conducted a family lineage analysis of dysferlinopathy, which enriched the variant spectrum of the DYSF gene and deepened the understanding of the clinical features, genetic characteristics, and diagnostic options of dysferlinopathy. In addition, this study also determined the pathogenicity of the mutation in this gene by an *in vivo* splicing assay and an *in vitro* minigene assay, which provides a reference for the clinical genetic diagnosis of dysferlinopathy.

## Materials and methods

### Subjects

**FIGURE 1:**
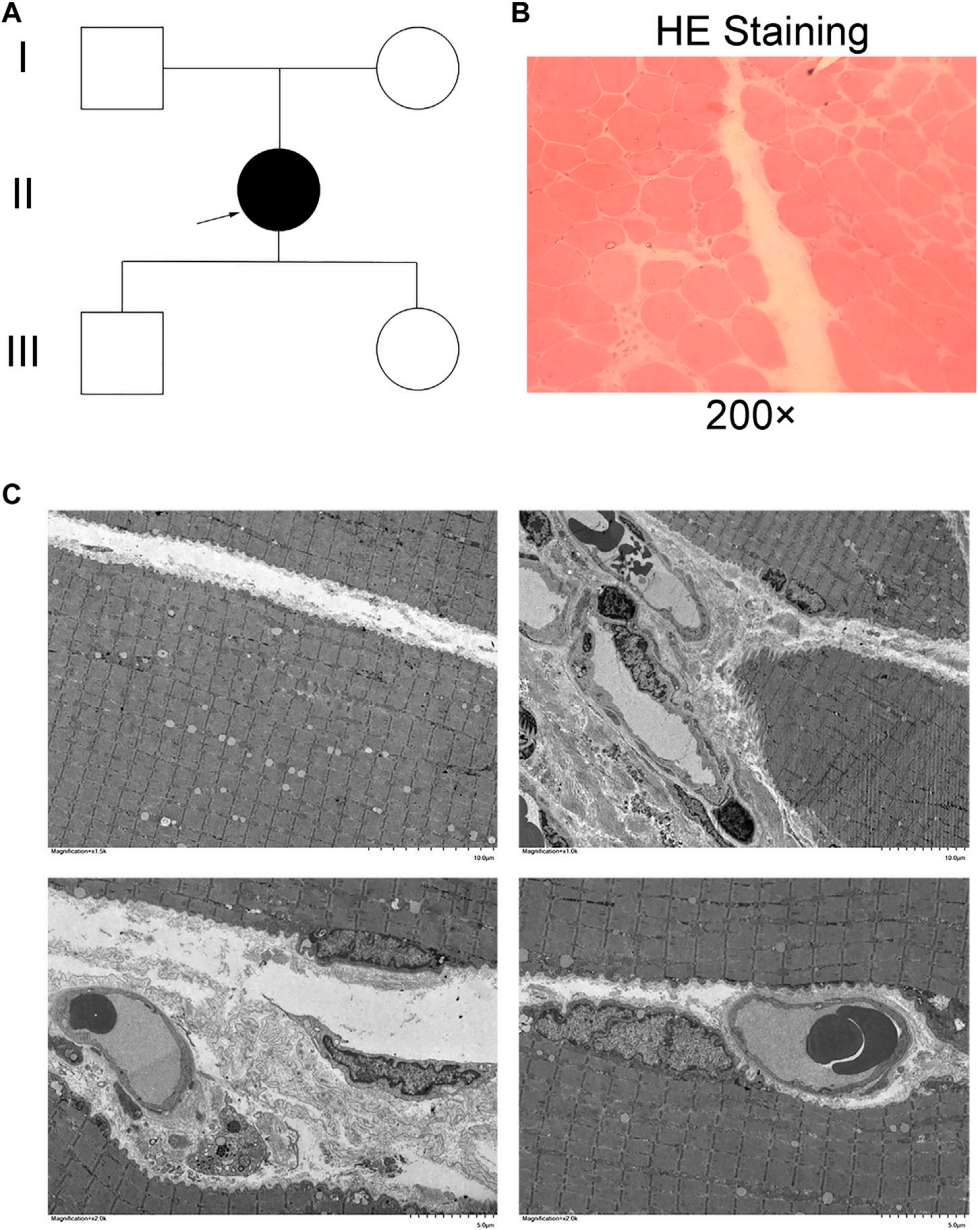
Clinical phenotype. **(A)** Pedigree of a three-generation family with dysferlinopathy (DYSF). Filled symbols represent affected individuals; open symbols represent unaffected individuals; squares depict men, and circles depict women. The proband is indicated by an arrow. **(B)** HE staining in muscle fibers. **(C)** Electron microscopy of the skeletal muscle.

The DYSF-affected family was referred to the Renmin Hospital of Wuhan University for weakness in both lower extremities and swollen calves and requested genetic testing for all family members. All the individuals were clinically assessed by at least two experienced clinicians, and a detailed questionnaire was completed to clarify the diagnosis and understand the genetic background. The diagnosis was based on a histological examination of the right biceps, muscle enzyme histochemical examination, femoris skeletal muscle electron microscopy, gene testing, and family history. Five individuals were tested in the DYSF-affected family, including one individual who was diagnosed with dysferlinopathy. In order to rule out the possibility that the variant was unique to the region, 100 local residents were recruited for alternative allele frequency testing in 2022. The lower extremities and calves of the control group appear normal. All participants recruited in this study provided informed consent for the study approved by the ethics committee of the Renmin Hospital of Wuhan University. The pedigree is shown in Figure 1.

### Whole-exome sequencing analysis

The genomic DNA was analyzed using target region capture high-throughput sequencing. The method was based on collecting DNA samples from blood, saliva, or other tissues; interruption of genomic DNA; and library preparation. The genomic DNA was interrupted, the library was prepared, and the DNA in the exons and adjacent splicing regions of the target gene was captured and enriched by a liquid-phase microarray. Finally, the variation was detected by a high-throughput sequencing platform.

The raw sequencing data were assessed for sequencing quality using AfterQC, and low-quality and splice-contaminated reads were removed. The filtered data were sequenced against the human hg19 reference genome using Burrows–Wheeler Aligner (BWA) software, and the capture effect was evaluated. SNVs (single-nucleotide variants) and Indels (insertions and deletions) in the genome were analyzed using Genome Analysis Toolkit (GATK) software. Subsequently, the 1,000 Genomes (a 1,000-human genomes dataset), Genome Aggregation Database (Genome AD) 2.1.1, and Exome Aggregation Consortium (ExAC) datasets were used to analyze the SNVs and Indels.

The SNVs and Indels were screened. The dbNSFP database was used to predict the pathogenicity of missense and splicing mutations. The Online Mendelian Inheritance in Man (OMIM) database, the Human Gene Mutation Database (HGMD), and the ClinVar database were used to screen for reported mutations. All mutant loci were classified using the American College of Medical Genetics and Genomics (ACMG) Genetic Variant Classification criteria and guidelines. Finally, all possible pathogenic loci were validated by Sanger sequencing.

### Bioinformatic analysis of variants

Different complementary online software tools were employed for splicing prediction: Human Splicing Finder (HSF) Version 2.4.1 (http://www.umd.be/HSF/), SpliceAI (https://spliceailookup.broadinstitute.org/), and varSEAK (https://varseak.bio/).

NM_001130987.1 was used as the reference sequence for mutation nomenclature, and DYSF exons were numbered according to the historical numbering used by the DYSF community.

Protein sequence alignments were performed using PolyPhen-2 (http://genetics.bwh.harvard.edu/pph2/). The following population or disease databases were examined to check allele frequencies: the Genome Aggregation Database gnomAD (https://gnomad.broadinstitute.org/), dbSNP (https://www.ncbi.nlm.nih.gov/snp/), TOPMed (https://www.nhlbiwgs.org/), Human Mutation Database (http://www.hgmd.cf.ac.uk), ClinVar (https://www.ncbi.nlm.nih.gov/clinvar/), and LOVD (https://www.lovd.nl/). Variant interpretation was based on ACMG criteria.

### Splicing assay *in vivo*

Normal human samples were used as a control for the mutations c.5628C>A p. D1876E and c.5633A>T p. Y1878F in the DYSF gene, and the mutant samples were tested to see if the mutation affected mRNA splicing. RNA was extracted using a blood total RNA extraction kit. Extracted RNA underwent reverse transcription to cDNA using the Hifair^®^ VnCov Multiplex One Step RT-qPCR Probe Kit (YEASEN, Shanghai, China). Nested amplification of normal patient samples was conducted with primers DYSF-5148-F1 (CTGTGGACTCCCACAGACCTACTGT)/DYSF-6167-R1 (TCC​TCA​AGC​TTA​GGG​TTC​ATG​TTG​G) and DYSF-5266-F2 (CGGACAGACCGTGTAATGTTTCAGG)/DYSF-5983-R2 (ACC​ATT​CTG​GGT​GGA​AAG​CAT​CAT​C). The PCR products were subjected to Sanger sequencing.

### Splicing assay of two novel variants of the DYSF gene and a single mutation of the DYSF gene by an *in vitro* minigene assay

PCR fragments, including the exon adjacent to each DYSF variant and at least 100 bp of the upstream and downstream introns, were amplified from the patient’s genomic DNA and cloned into the pcDNA3.1 vector and pcMINI-C vector. Exon49 (89bp) -intron49 (269 bp) -Exon50 (96 bp) -intron50 (397 bp) -Exon51 (142 bp) of DYSF was inserted into the pcDNA3.1 vector. Part of intron49 (251 bp) -Exon50 (96 bp) -intron50 (397 bp) -Exon51 (142 bp) of DYSF was inserted into the pcMINI-C vector. The correctness of each vector was verified by direct Sanger sequencing. HEK293 cells (6 × 10^5^) and HeLa cells were transfected with 1 µg of the wild type or the mutated minigene using Hieff Trans Liposomal Transfection Reagent (YEASEN, Shanghai, China) according to the manufacturer’s instructions. After 48 h, total RNA was extracted with TRIzol™ (ThermoFisher Scientific, Waltham, MA, United States) and retrotranscribed using the Hifair^®^ V nCov Multiplex One Step RT-qPCR Probe Kit (YEASEN, Shanghai, China). The resulting cDNA was then amplified using specific primers for the DYSF gene, designed on exon 49 (forward) and exon 50 (reverse), in order to avoid the amplification of eventual ectopically expressed DYSF transcripts. PCR products were separated on a 1.5% agarose gel, and individual bands were excised and sequenced using amplification primers. The purified bands were subcloned using a TOPO TA Cloning kit when required (ThermoFisher Scientific, Waltham, MA, United States), and single clones were sequenced. Densitometric analyses were performed with the ImageJ software (https://imagej.nih.gov/ij/).

The vector construction strategy, RNA extraction method, reverse transcription, and PCR amplification methods of a single mutation of the DYSF gene by *in vitro* minigene assay were similar to those used for two variants of the DYSF gene by *in vitro* minigene assay, with only a single mutation instead of a double mutation.

## Results

### Clinical phenotype

A pedigree of the three-generation family that participated in this study was constructed, and it included one family member affected by DYSF and four unaffected family members (Figure 1A). The proband II was a 42-year-old women who presented with weakness of the lower limbs for 2 years and edema of the lower leg. Histological examination of the right biceps femoris muscle showed that the diameter of the muscle fibers ranged from 40 to 95 microns. The diameter of the muscle fibers varied, and the muscle fibers included circular, small, round, small horn atrophic muscle fibers, hypertrophic muscle fibers, necrosis, and regeneration muscle fibers. There was no obvious tissue hyperplasia or inflammatory cell infiltration around blood vessels or between muscle fibers (Figure 1B). Electron microscopy of the skeletal muscle revealed a locally residual, curled basal membrane of muscle cells from necrotic or highly atrophic muscle cells. A few muscle cell spaces widened, and a few blood vessel base membranes exhibited thickened, stratified, focal small vessel aggregation with collagen fibril deposition (Figure 1C). The above results confirmed that the patient had muscular dystrophy phenotypes such as weakness of lower limbs and edema of lower legs, which needed further diagnosis combined with genetic testing.

### Genetic analysis of whole-exome sequencing

**FIGURE 2:**
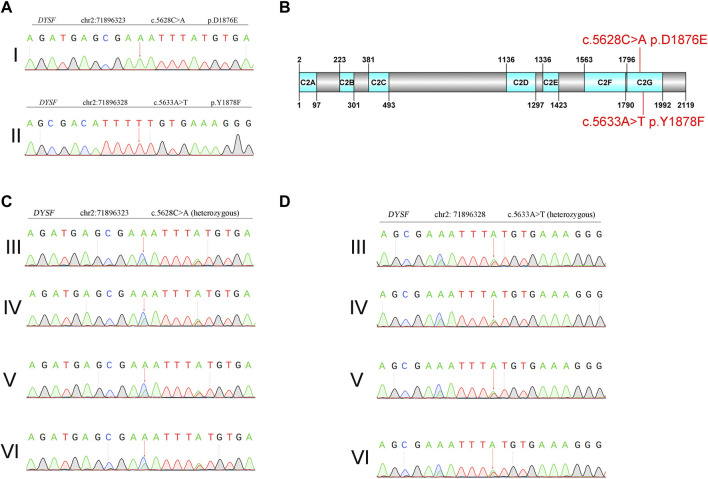
Genetic analysis of Sanger sequencing. **(A)** Proband. I: c.5628C>A p. D1876E. Proband. II: c.5633A>T p. Y1878F. **(B)** Domain organization of dysferlin. **(C)** and **(D)** Sanger sequencing of family members. III: mother of the proband. IV: father of the proband. V: son of the proband. VI: daughter of the proband.

Whole-exome sequencing using the genomic DNA of proband II (Figure 1A) was performed. In total, 850 million uniquely mapped reads with MAPQ ≥30 were generated, covering 97.36% of exome target regions at least 20×. The number of genes captured in the target area was 2,980. The total length of the target region for capture sequencing was 7,534,869 bp. The coverage percentage of sequencing data in the target region was 99.92%. The average depth of sequencing data in the target region was 104.04. No pathogenic or suspected pathogenic mutation was detected that was consistent with the patient’s phenotype. Two homozygous mutations with insufficient evidence of pathogenicity, but a good match with the patient’s phenotype, do not exclude a possible pathogenic mutation: DYSF c.5628C>A p. D1876E and c.5633A>T p. Y1878F (Figure 2A).

The variant was not present in the Human Gene Mutation Database (http://www.hgmd.cf.ac.uk/ac/), HPSD (http://liweilab.genetics.ac.cn/HPSD/), or the dbSNP (http://www.ncbi.nlm.nih.gov/SNP/), nor was it present in the DNA samples from 100 normal samples or in the control databases (gnomAD, http://gnomad-sg.org/). The variant was located in the C-terminal signaling domain as the C2G domain of the dysferlin protein (Figure 2B). The mother, father, son, and daughter of the proband had cis compound heterozygous mutations of DYSF (Figures 2C,D). The pathogenicity ratings of both mutations in DYSF, according to the ACMG guidelines, were variants of uncertain significance (VUSs). These results confirmed that two homozygous mutations in DYFS may be the cause of dysferlinopathy, and dysferlinopathy was inherited by autosomal recessive inheritance.

### Bioinformatic analysis of variants

We performed an *in silico* analysis to predict the possible effects of variants on splicing (Table 1). The c.5628C>A mutation of DYSF was located at position −15 of exon 50. Three bioinformatics databases were used to predict how the mutation would affect splicing. HSF suggested that the mutation had no significant impact on splicing signals; varSEAK and SpliceAI suggested that the mutation had no splicing effect. The mutation c.5633A>T of DYSF was located at position −10 of Exon 50. HSF suggested that the mutation altered auxiliary sequences and made a significant alteration of the ESE/ESS motif ratio (−6). VarSEAK and SpliceAI also suggested that the mutation had no splicing effect. Mutations c.5628C>A and c.5633A>T of DYSF were not included in the gnomAD database and may be new. The Mutation Taster database indicated that c.5628C>A and c.5633A>T of DYSF were predicted to be “disease-causing” mutations that could change amino acid sequences, affect protein features, and may have an effect on splicing, which leads to dysferlinopathy.

**TABLE 1:** Bioinformatics analysis of DYSF variants.

Genomic coordinate	cDNA change	Effect on protein	Location	Human splicing finder	VarSEAK	SpliceAI
Chr2:71896323	c.5628C>A	p.D1876E	Exon 50	No significant impact on splicing signals	No splicing effect	No splicing effect
Chr2:71896328	c.5633A>T	p. Y1878F	Exon 50	Alteration of auxiliary sequences	No splicing effect	No splicing effect

### Mutations affected splicing *in vivo*

**FIGURE 3:**
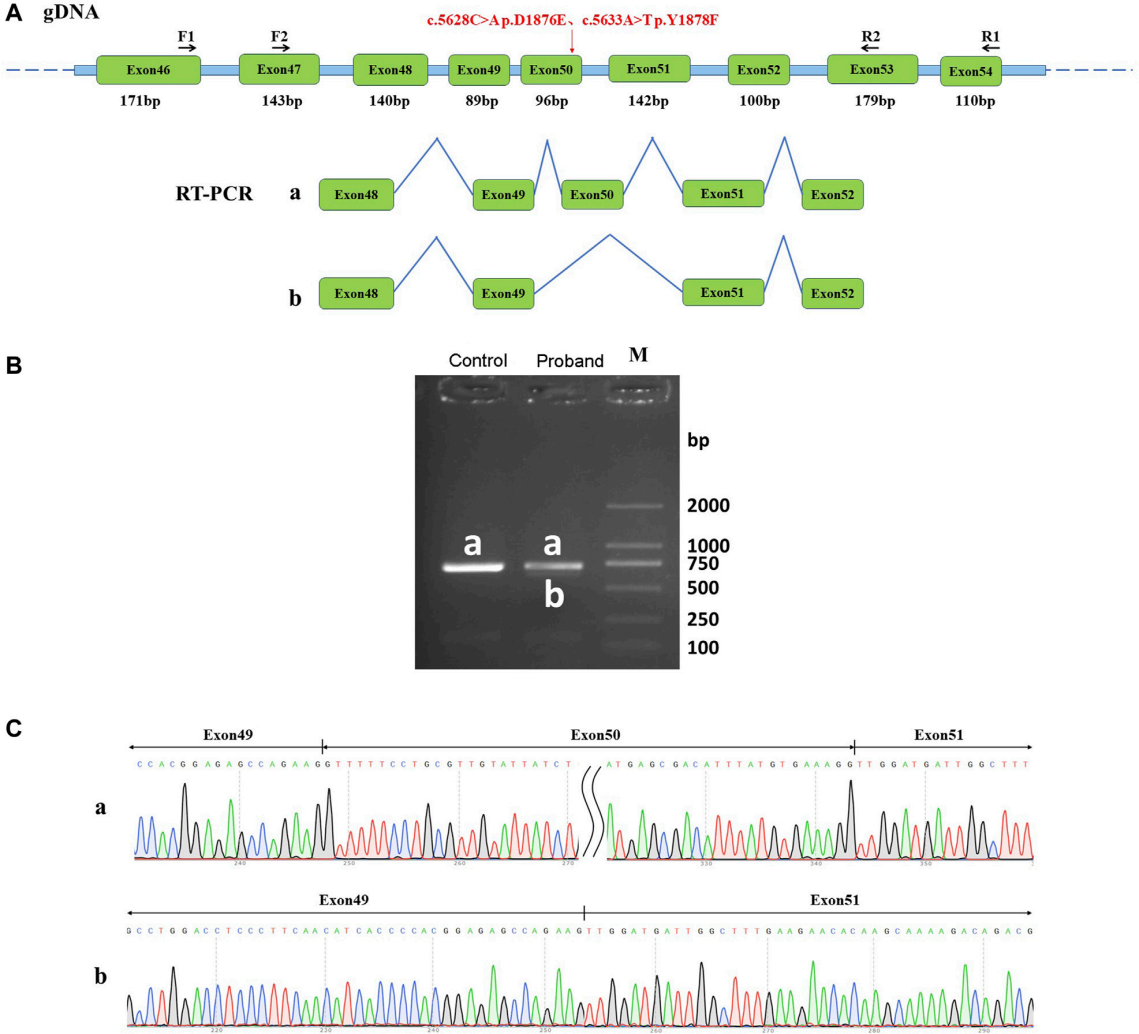
Effect of mutation on mRNA shearing. **(A)** Schematic of primer design and shearing diagram, in which the red arrow points to the position of the mutation; **(B)** Agarose gel electrophoresis of RT-PCR products, naming both normal shear bands of normal human samples and the proband samples as band a, and abnormal shear bands of the patient samples as band b; **(C)** Graph of sequencing results corresponding to the shear bands a and b.

Sanger sequencing results showed that the normal human had only one band, named band a, which was a normal splicing band with Exon48 (140 bp)-Exon49 (89 bp)-Exon50 (96 bp)-Exon51 (142 bp)-Exon52 (100 bp). The patient had two bands, named band a and band b. Patient band a was a normal splicing band with Exon48 (140 bp)-Exon49 (89 bp)-Exon50 (96 bp)-Exon51 (142 bp)-Exon52 (100 bp). Patient band b was an abnormal splicing band with Exon48 (140 bp)-Exon49 (89 bp)-Exon51 (142 bp)-Exon52 (100 bp), in which Exon50 jumps (Figures 3A–C). *In vivo* splicing assays showed that the two homozygous mutations c.5628C>A p. D1876E and c.5633A>T p. Y1878F affected the normal splicing of mRNA. Two types of splicing exist after the mutation: normal splicing and Exon50 jumping.

The mutation resulted in an Exon50 jump, which was expressed at the cDNA and protein levels as c.5547_5642del p. Arg1849_Gly1881delinsSer. The Exon50 jump did not result in a subsequent reading frame change and only resulted in a 32-aa deletion within the protein. These results revealed that two adjacent missense mutations of DYSF could affect the normal splicing function of mRNA *in vitro*, thus leading to the development of dysferlinopathy.

### Two novel homozygous mutations of DYSF affected splicing *in vitro*

**FIGURE 4:**
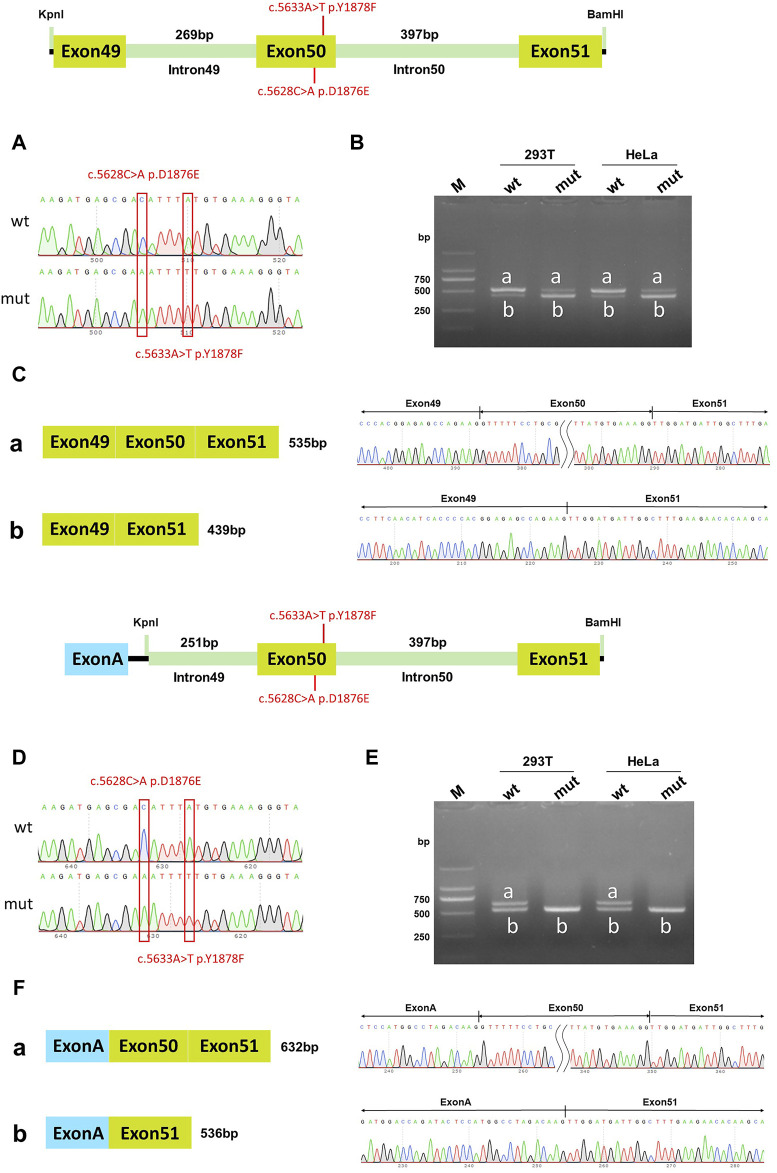
pcDNA3.1 vector and pcMINI-C vector detection results. **(A)** Minigene construction sequencing map in pcDNA3.1 vector, wt at the top, c.5628C>A p. D1876E and c.5633A>T p. Y1878F mut at the bottom; **(B)** RT-PCR transcription analysis of agarose gel electrophoresis in the pcDNA3.1 vector; the bands in HeLa and 293T cells are labeled a and b; **(C)** Minigene splicing diagram in pcDNA3.1 vector; **(D)** Minigene construction sequencing map in pcMINI-C vector, wt at the top, c.5628C>A p. D1876E and c.5633A>T p. Y1878F mut at the bottom; **(E)** RT-PCR transcription analysis of agarose gel electrophoresis in pcMINI-C vector; the bands in HeLa and 293T cells were labeled a and b; **(F)** Minigene splicing diagram in pcMINI-C vector. Red * indicates the mutation position.

The RT-PCR results showed that the wild-type DYSF gene in HeLa and 293T cells had two bands, of which the larger band was 632bp and named band a, and the smaller band was named band b. The mutation in the DYSF gene in HeLa and 293T cells also had two bands, named mutant band a and mutant band b. The sequencing results showed that the wild-type band a was a normal splicing strip with the splicing mode of Exon49 (89bp)-Exon50 (96bp)-Exon51 (142bp) in pcDNA3.1 vector; the wild-type band b was an abnormal splicing strip with an Exon50 jump and the splicing mode of Exon49 (89bp)-Exon51 (142bp). The mutant band a was a normal splicing strip with the splicing mode of Exon49 (89bp)-Exon50 (96bp)-Exon51 (142bp). The mutant band b was an abnormal splicing band with Exon50 jumps, and the splicing mode was Exon49 (89bp)-Exon51 (142bp) (Figures 4A–C). The wild type was dominated by band a, while the mutant type was dominated by band b. The same results were observed in the pcMINI-C vector (Figures 4D–F). The mutation resulted in cDNA and protein levels being the same as the *in vivo* splicing assay. These results were consistent with the *in vivo* splicing assay, suggesting that two homozygous mutations affected the DYSF gene's normal splicing function.

### The c.5628C>A p.D1876E single mutation in DYSF affected *in vitro* splicing, while c.5633A>T p.Y1878F did not

**FIGURE 5:**
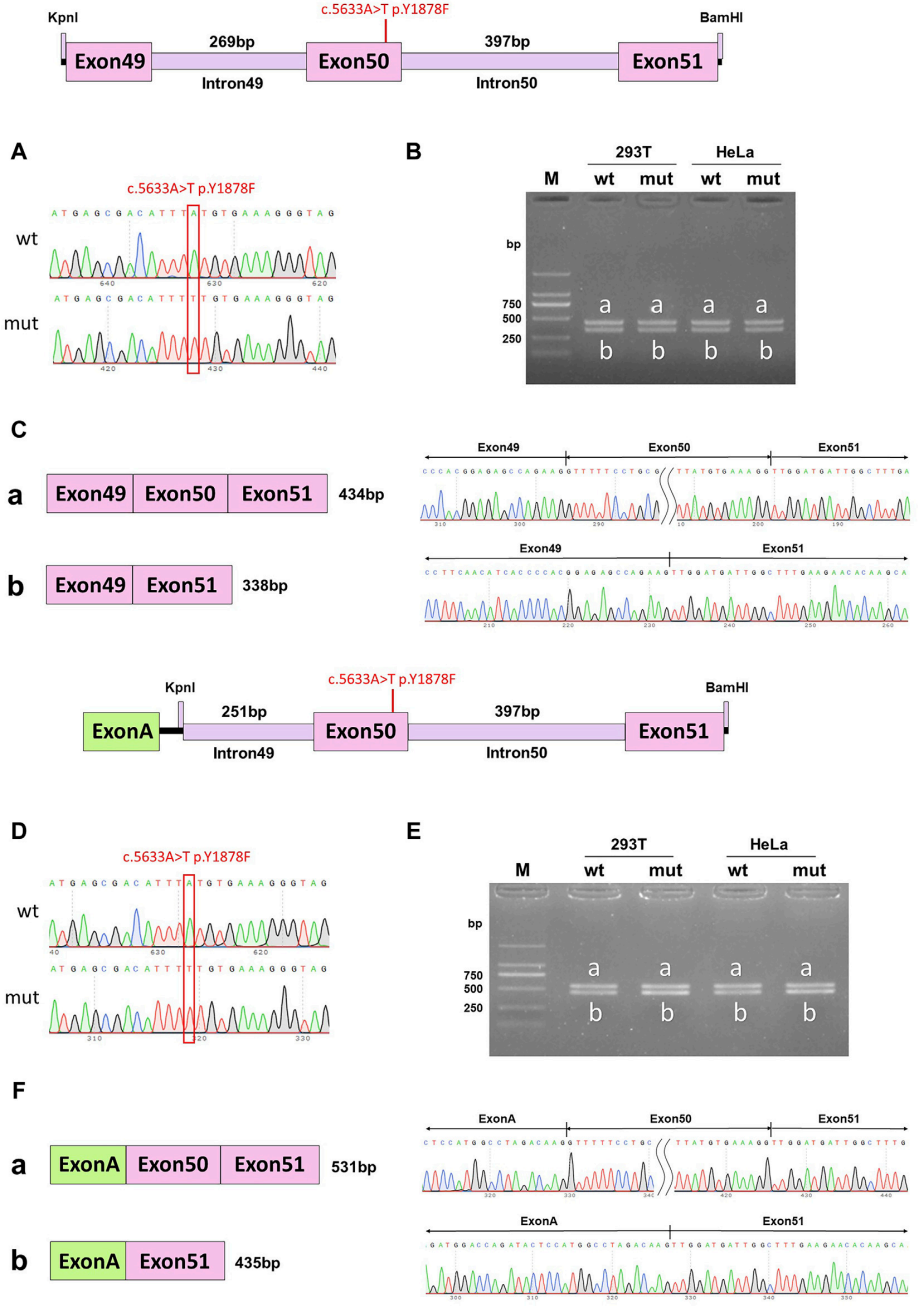
pcDNA3.1 vector and pcMINI-C vector detection results. **(A)** Minigene construction sequencing map in pcDNA3.1 vector, wt at the top, c.5633A>T p. Y1878F mut at the bottom; **(B)**. RT-PCR transcription analysis of agarose gel electrophoresis in pcDNA3.1 vector; the bands in HeLa and 293T cells are labeled a and b; **(C)** Minigene splicing diagram in the pcDNA3.1 vector; **(D)**. Minigene construction sequencing map in the pcMINI-C vector, wt at the top, ce.5633A>T p. Y1878F mut at the bottom; **(E)** RT-PCR transcription analysis of agarose gel electrophoresis in the pcMINI-C vector; the bands in HeLa and 293T cells are labeled a and b; **(F)** minigene splicing diagram in the pcMINI-C vector. Red * indicates the mutation position.

Minigene experiments with single mutations were conducted to further verify which mutation of DYSF affected the splicing function. In the wild-type pcDNA3.1 vector, band a was a normal splicing band, and the splicing mode was ExonA (192bp)-Exon50 (96bp)-Exon51 (142bp); wild-type band b was an abnormal splicing band, with Exon50 jumping, and the splicing mode was ExonA (192bp)-Exon51 (142bp). The c.5633A>T p. Y1878F mutant band a was a normal splicing band, and the splicing mode was ExonA (192bp)-Exon50 (96bp)-Exon51 (142bp); mutant band b was an abnormal splicing band, Exon50 jumps, and the splicing mode was ExonA (192bp)-Exon51 (142bp) (Figures 5A–C). The same results were observed in the pcMINI-C vector (Figures 5D–F). Both the wild-type and mutant splice patterns consisted of bands a and b, and there was no significant change in the ratio of bands a and b. These results indicated that the mutation c.5633A>T p. Y1878F did not affect the normal splicing of the mRNA.

**FIGURE 6:**
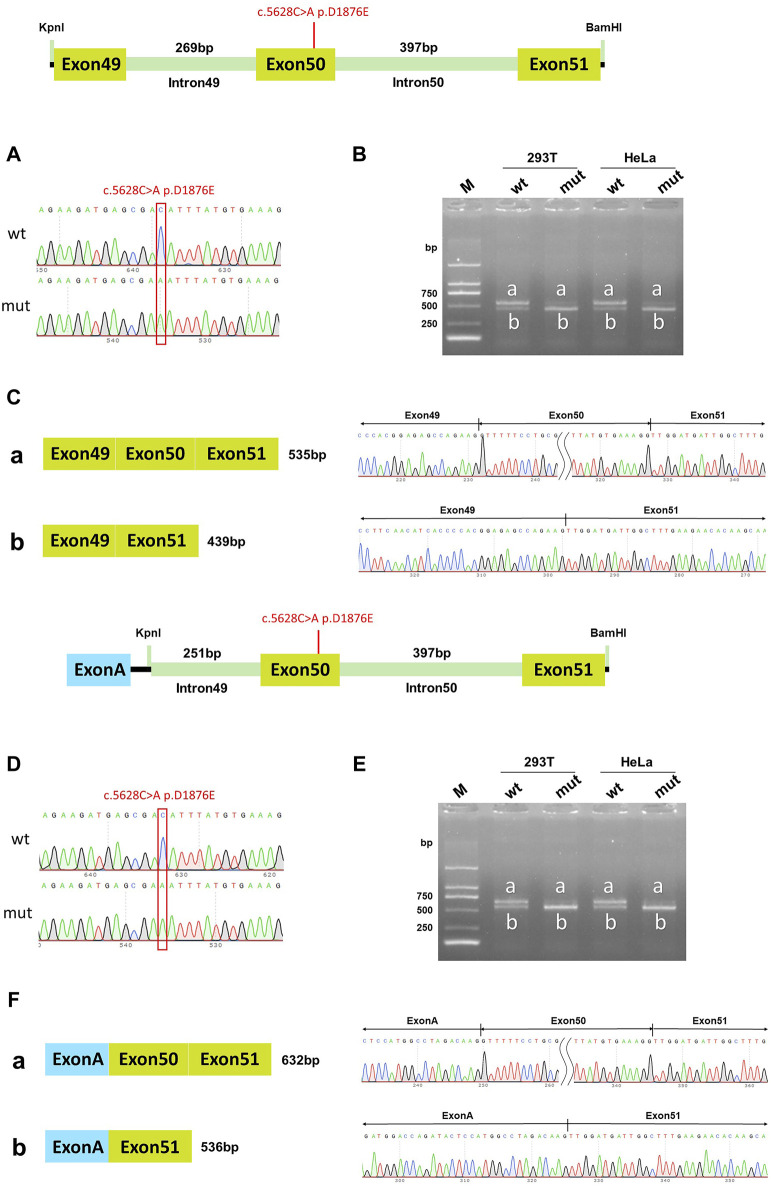
pcDNA3.1 vector and pcMINI-C vector detection results. **(A)** Minigene construction sequencing map in the pcDNA3.1 vector, wt at the top, c.5628C>A p. D1876E mut at the bottom; **(B)**. RT-PCR transcription analysis of agarose gel electrophoresis in the pcDNA3.1 vector; the bands in HeLa and 293T cells are labeled a and b; **(C)** Minigene splicing diagram in the pcDNA3.1 vector; **(D)** Minigene construction sequencing map in the pcMINI-C vector, wt at the top, c.5628C>A p. D1876E mut at the bottom; **(E)** RT-PCR transcription analysis of agarose gel electrophoresis in the pcMINI-C vector; the bands in HeLa and 293T cells are labeled a and b; **(F)** Minigene splicing diagram in pcMINI-C vector. Red * indicates the mutation position.

In the wild-type pcDNA3.1 vector, band a was a normal splicing band, and the splicing mode was Exon49 (89bp)-Exon50 (96bp)-Exon51 (142bp); wild-type band b was an abnormal splicing band with Exon50 jumping, and the splicing mode was Exon49 (89bp)-Exon51 (142bp). The c.5628C>A p. D1876E mutant band a was a normal splicing band, and the splicing was Exon49 (89bp)-Exon50 (96bp)-Exon51 (142bp); mutant band b was an abnormal splicing band with Exon50 jumps, and the splicing mode was Exon49 (89bp)-Exon51 (142bp) (Figures 6A–C). The same results were also observed in the pcMINI-C vector (Figures 6D–F). The wild-type splice pattern was predominantly characterized by band a, while the mutant type was predominantly characterized by band b. These results showed that mutation c.5628C>A p. D1876 affected the normal splicing of gene mRNA, and the pcMINI-C results were consistent with the pcDNA3.1 results. There were two kinds of variable splicing after the mutation: one was normal splicing, which accounts for a small percentage, and the other was Exon50 jumping, which accounts for a large percentage.

## Discussion

Dysferlinopathy is an autosomal recessive inherited skeletal muscle disorder caused by a mutation in DYSF, resulting in abnormal dysferlin protein expression (Zhang et al., 2022). Characterized by weakness and atrophy of the proximal pelvic–femoral muscles, it develops in adolescence or early adulthood with a massive increase in serum creatine kinase levels and progresses slowly. The dysferlin protein has a role in repairing damaged skeletal muscle cell membranes, which can cause degenerative necrosis under physiological conditions when the membranes are fractured by mechanical stress and are not repaired in time (Pesciotta et al., 2014). DYSF mutations could affect the repair function of the cell membrane after physiological damage, thus affecting the stability of the cell membrane. The membrane repair process could trigger an inflammatory cascade reaction, resulting in irreversible damage to myofibers (Bittel et al., 2020).

Clinical diagnosis mostly relies on laboratory ancillary tests and genetic testing, including serological markers of myocyte membrane disruption, CK (significant 35- to 200-fold increase in CK in blood); electromyography suggesting myogenic changes; pathological examination suggesting typical myotonic manifestations, inflammatory response, and myocyte membrane dysferlin protein deficiency (Fanin and Angelini, 2016). Genetic testing for DYSF mutations is the gold standard for clinical diagnosis, and Western blot testing for dysferlin deficiency in monocytes, combined with clinical manifestations, can also diagnose dysferlinopathy (Shin et al., 2015).

Although many scholars have reported mutations in several loci of DYSF (Spuler et al., 2008; Santos et al., 2010), the two mutations c.5628C>A p. D1876E and c.5633A>T p. Y1878F of DYSF involved in this study have not yet been reported. Validating the effects of the two *de novo* mutations in DYSF on dysferlin protein expression, localization, structure, and function *in vivo* and *in vitro* was important to reveal the pathogenic mechanism of dysferlinopathy.

In this study, the proband (II) was a 42-year-old woman with weakness of the lower limbs for 2 years and edema of the lower leg. Dysferlinopathy includes a spectrum of muscle diseases characterized by three major phenotypes: MMD, LGMD2B, and DMAT (Aoki et al., 2004). MMD (median age of onset 19 years) is characterized by muscle weakness and atrophy, most marked in the distal parts of the legs, especially the gastrocnemius and soleus muscles (Majumdar et al., 2021). The clinical phenotype in this study had the characteristics of the MMD type. Histological examination of the right biceps femoris suggested myofibrosis. Muscle enzyme histochemical examination showed that acid phosphatase (acid) activity increased. A small amount of mitochondrial aggregation was observed under the membrane of NADH-TR, SDH, and COX. Electron microscopy of the skeletal muscle revealed a small amount of necrosis of muscle cells. These results confirmed that proband (II) had muscular dystrophy phenotypes such as weakness of lower limbs and edema of lower legs, muscular dystrophy, and necrosis of muscle cells. These phenotypes have not been reported in the previous literature, and this mutation may provide evidence of a correlation between mutations in the DYSF gene and the dysferlinopathy phenotype.

In order to clarify the diagnosis of the disease, whole-exome sequencing and Sanger sequencing were performed to validate the prior observations. The results showed the presence of DYSF c.5628C>A p. D1876E and c.5633A>T p. Y1878F mutations in the proband, which matched the patient’s phenotype but lacked sufficient evidence to suggest that the patient’s mutations were disease-causing mutations. The mother, father, son, and daughter of the proband have cis compound heterozygous mutations in DYSF. In contrast, the proband patient had doubly homozygous mutations in two different loci of DYSF, a relatively rare inheritance pattern. These results confirmed that the two homozygous mutations of DYFS may be the cause of dysferlinopathy and that dysferlinopathy was inherited in an autosomal recessive manner, and this study further enriches the genetic spectrum of the DYSF gene.

In order to explore the pathogenic mechanism of two novel mutations of DYSF, we first conducted bioinformatics prediction and analyzed the influence of double mutation on mRNA splicing. The HSF, Varseak, and SpliceAI bioinformatics databases suggested that the c.5628C>A DYSF mutation had no significant impact on splicing signals. HSF suggested that the c.5633A>T mutation altered auxiliary sequences and significantly altered the ESE/ESS motif ratio (−6). Varseak and SpliceAI suggested that the c.5633A>T mutation had no splicing effect. Query results about these mutations showed that these mutations were not included in the gnomAD database and may be new. The Mutation Taster database predicted that c.5628C>A and c.5633A>T of DYSF were disease-causing mutations and may affect splicing. These results suggested that two novel missense mutations of DYSF might affect splicing, leading to dysferlinopathy.

Alternative splicing can generate different isoforms of one single protein with different enzymatic activity, substrate specificity, subcellular localization, or an altered ability to interact with other proteins or DNA, which not only contributes to proteome diversity but also has a regulatory function in many physiological processes (Majumdar et al., 2021). To investigate the pathogenesis of the two DYSF homozygous mutations, *in vivo* studies of their effect on the splicing function of pre-mRNA were conducted. The results indicated that the two homozygous mutations in the DYSF gene affected splicing function and may be one of the causes of dysferlinopathy.

The establishment of *in vitro* cell models of genetic defects is conducive to the experimental study of cell function before and after mutation and to the study of pathological genetic mechanisms of diseases (Padgett et al., 1986). Proper splicing of mRNA precursors is a key step in eukaryotic gene expression, and abnormal splicing can affect gene expression and function (Liang et al., 2023). The minigene technique is the *in vitro* verification experiment of abnormal mRNA splicing, aimed at the cellular-level verification of the influence of mutation sites on mRNA splicing (Cooper, 2005) by cloning the target genomic fragment, constructing a recombinant expression vector, transfecting the cell line, collecting the cells to extract RNA, and then performing and cDNA inversion to verify whether the mutation affects mRNA splicing (van der Klift et al., 2015). It has been reported that minigene experimental results have an almost 100% accuracy in predicting mutation pathogenicity and whether it affects splicing. In order to confirm the effects of two DYSF mutations on splicing function, two mutant vectors of DYSF gene c.5628C>A and c.5633A>T were constructed and transfected to HEK293 and HeLa cells. The sequencing results showed the mutation resulted in an Exon50 jump and a 32-aa deletion within the protein. These results revealed that two adjacent missense mutations of DYSF could affect the normal splicing function of mRNA *in vitro*, thus leading to the development of dysferlinopathy.

Vectors for individual mutations were constructed separately to confirm whether the effect of mutations on the splicing function was a function of the double mutation as a whole or a single mutation. The results indicated that the c.5633A>T p. Y1878F mutant band a was a normal splicing band, and mutant band b was an abnormal splicing band with Exon50 jumps. Both the wild-type and mutant splice patterns consisted of bands a and b. The mutation c.5633A>T p. Y1878F did not affect the normal splicing of the mRNA. The c.5628C>A p. D1876E mutant band a was a normal splicing band, and mutant band b was an abnormal splicing band with Exon50 jumps. The wild-type splice pattern was predominantly characterized by band A, while the mutant type was predominantly characterized by band B. The mutation c.5628C>A p. D1876 affected the normal splicing of gene mRNA. The different percentages of the two bands may be one of the reasons that the mutation affected the splicing function. A single mutation (c.5628C>A p. D1876) and two homozygous mutations (c.5628C>A and c.5633A>T) affected the splicing function, while another single mutation (c.5633A>T) and cis compound heterozygous mutations did not affect splicing. This was related to the autosomal recessive mode of inheritance of DYSF, where compound heterozygous mutations did not have a phenotype, and only homozygous mutations could have a phenotype.

In summary, this study demonstrates for the first time that DYSF double mutations were associated with the development of dysferlinopathy. Bioinformatics predicted that the mutations affected the normal splicing function of mRNA. *In vivo* splicing experiments confirmed the presence of the double mutation on the normal splicing function of mRNA. *In vitro* minigene analysis confirmed that the c.5628C>A p. D1876E mutation affected splicing function and might be a cause of dysferlinopathy.

## Data Availability

The original data presented in the study is publicly available. This data can be found here: Genome Sequence Archive for human database (GSA-human). Accession number: HRA007508 [https://ngdc.cncb.ac.cn/gsa-human/browse/HRA007508].
